# DENSE-SIM: A modular pipeline for the evaluation of cine displacement encoding with stimulated echoes images with sub-voxel ground-truth strain

**DOI:** 10.1016/j.jocmr.2025.101866

**Published:** 2025-02-21

**Authors:** Hugo Barbaroux, Michael Loecher, Yannick Brackenier, Karl P. Kunze, Radhouene Neji, Dudley J. Pennell, Daniel B. Ennis, Sonia Nielles-Vallespin, Andrew D. Scott, Alistair A. Young

**Affiliations:** a School of Biomedical Engineering and Imaging Sciences, King’s College London, London, UK; b Cardiovascular Magnetic Resonance Unit, Royal Brompton Hospital (Guy’s and St Thomas’s NHS Foundation Trust), London, UK; c Department of Radiology, Stanford University, Stanford, California, USA; d MR Research Collaborations, Siemens Healthcare Limited, Camberley, UK; e National Heart and Lung Institute, Imperial College London, London, UK; f Division of Radiology, Veterans Affairs Health Care System, Palo Alto, California, USA

**Keywords:** Cardiac, MRI, Strain, DENSE, Image synthesis

## Abstract

**Background:**

Myocardial strain is a valuable biomarker for diagnosing and predicting cardiac conditions, offering additional prognostic information to traditional metrics such as ejection fraction. While cardiovascular magnetic resonance (CMR) methods, particularly cine displacement encoding with stimulated echoes (DENSE), are the gold standard for strain estimation, evaluation of regional strain estimation requires precise ground truth. This study introduces DENSE-SIM, an open-source simulation pipeline for generating realistic cine DENSE images with high-resolution known ground-truth strain, enabling evaluation of accuracy and precision in strain analysis pipelines.

**Methods:**

This pipeline is a modular tool designed for simulating cine DENSE images and evaluating strain estimation performance. It comprises four main modules: 1) anatomy generation, for creating end-diastolic cardiac shapes; 2) motion generation, to produce myocardial deformations over time and Lagrangian strain; 3) DENSE image generation, using Bloch equation simulations with realistic noise, spiral sampling, and phase cycling; and 4) strain evaluation. To illustrate the pipeline, a synthetic dataset of 180 short-axis slices was created and analyzed using the commonly used DENSEanalysis tool. The impact of the spatial regularization parameter (k) in DENSEanalysis was evaluated against the ground-truth pixel strain, to particularly assess the resulting bias and variance characteristics.

**Results:**

Simulated strain profiles were generated with a myocardial signal-to-noise ratio (SNR) ranging from 3.9 to 17.7. For end-systolic radial strain, DENSEanalysis average signed error (ASE) in Green strain ranged from 0.04 ± 0.09 (true-calculated, mean ± std) for a typical regularization (k = 0.9), to −0.01 ± 0.21 at low regularization (k = 0.1). Circumferential strain ASE ranged from −0.00 ± 0.04 at k = 0.9 to −0.01 ± 0.10 at k = 0.1. This demonstrates that the circumferential strain closely matched the ground truth, while radial strain displayed more significant underestimations, particularly near the endocardium. A lower regularization parameter from 0.3 to 0.6 depending on the myocardial SNR would be more appropriate to estimate the radial strain, as a compromise between noise compensation and global strain accuracy.

**Conclusion:**

Generating realistic cine DENSE images with high-resolution ground-truth strain and myocardial segmentation enables accurate evaluation of strain analysis tools, while reproducing key in-vivo acquisition features, and will facilitate the future development of deep-learning models for myocardial strain analysis, enhancing clinical CMR workflows.

## Graphical abstract

**Figure:**
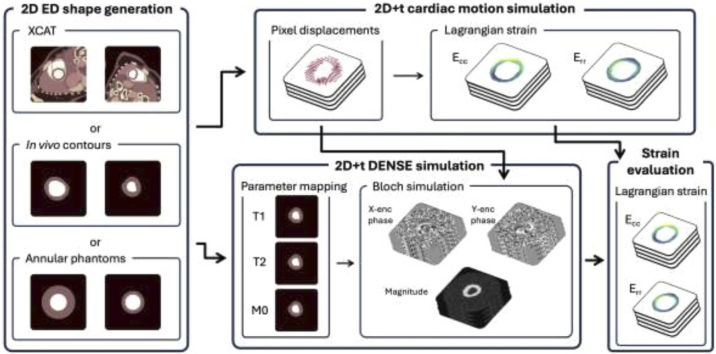

## 1 Background

Myocardial strain has been increasingly studied and validated as a biomechanical biomarker that provides added diagnostic and prognostic value for a range of cardiac pathologies [1], [2]. In particular, studies have shown additional prognostic information in strain over the standard left ventricular ejection fraction [3], [4]. Recent guidelines [5], [6], [7], [8] include global longitudinal strain from echocardiography as a recommendation. However, the need for highly trained operators for strain analysis in echocardiography, and the lack of robust accurate analysis methods for evaluating regional strain [9] has impeded the clinical utility of myocardial strain evaluation.

Cardiovascular magnetic resonance (CMR) methods are considered the gold standard for strain estimation and can be categorized into two groups: analysis-based and acquisition-based. Analysis-based methods such as feature tracking solely rely on a post-processing analysis to estimate strain, for example by estimating a displacement field between cine images from different time frames in the cardiac cycle [10]. However, extracting accurate regional strain remains a challenge [11]. Acquisition-based methods, such as CMR tagging [12], [13], use dedicated acquisition methods that provide additional sensitivity to or encoding of displacements into the imaging data. One of the most accurate acquisition sequences [11] is cine displacement encoding with stimulated echoes (DENSE) [14], [15], [16], [17], which encodes the displacement of tissue from a reference time point within the image phase.

Machine learning methods now enable automatic analysis of DENSE data [18], [19]. For example, Wang et al. [20] used DENSE to enable more accurate segmental strain estimation from feature tracking. However, the lack of a readily available, noninvasive, and accurate measure of regional strain to serve as a ground truth severely limits these automated analysis methods since manual validation may be biased. Further barriers to clinical translation are caused by the different strain results [21] obtained from different analysis methods, likely due to bias in the manually defined ground truth used to develop the techniques. The open-source DENSEanalyis Matlab tool [22], [23], currently the most widely used for clinical DENSE evaluations, requires the selection of regularization parameters to minimize noise effects at the same time as minimizing systematic errors due to oversmoothing. The regularization often limits the accuracy, in particular for radial strains [24].

Recent works have compared DENSE and tagged magnetic resonance using synthetic images with simplified geometries [24]. However, while deformation and image simulation methods have been proposed for cine [25] and tagging [26], [27] CMR, to our knowledge, there are no realistic DENSE simulation pipelines currently available.

In this study, we present DENSE-SIM, a novel open-source simulation pipeline for generating cine DENSE images with high-resolution (sub-voxel) ground-truth strain. This modular pipeline can generate two-dimensional (2D) short-axis cardiac deformations from a variety of myocardial contours and create associated realistic DENSE images from Bloch equation simulations, including phase cycling, partial volume effects, and spiral sampling artifacts. We qualitatively and quantitatively validate the features of the simulation pipeline in a virtual cohort of deformations and image simulations, and evaluate the DENSEanalysis tool to examine the effects of regularization with reference to ground-truth strains.

## 2 Methods

**Fig. 1:**
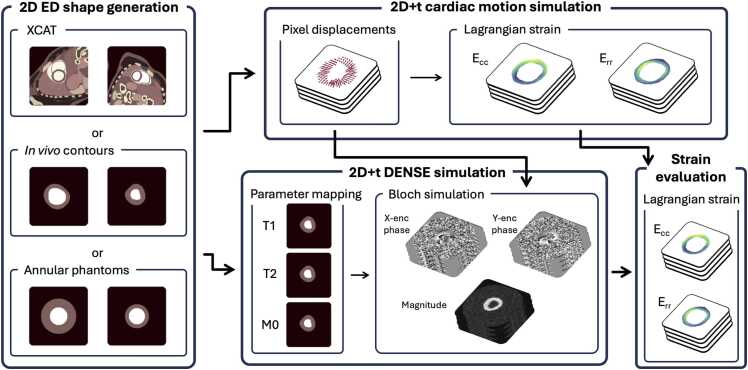
Illustration of the DENSE-SIM simulation pipeline. End-diastolic cardiac shapes and background tissues are generated (left) and then used as input to generate cardiac deformations through time (top right). A Bloch simulation generates DENSE images (bottom center) using the generated ground-truth displacements. (Bottom right) Enables the strain computation from the images and comparison with actual ground truth. *2D* two-dimensional, *ED* end-diastolic, *DENSE* displacement encoding with stimulated echoes, *XCAT* extended cardiac-torso, *Err* radial strain, *Ecc* circumferential strain

The DENSE-SIM pipeline is schematically summarized in Fig. 1. This pipeline consists of four parts: an anatomy generation module, to create cardiac shapes and surrounding tissues; a motion generation module, to generate pixel cardiac Lagrangian displacements through time; a DENSE image generation module, to generate cine DENSE images consistent with the created cardiac shapes and through-time myocardial displacements; and a strain evaluation module to compute strain from the simulated images and compare with the known ground truth.

This pipeline is intended to be a general modular tool for DENSE image generation, not constrained to specific simulation methods. While the implementation described and validated here was made by developing specific methods for each module to produce realistic images, this work is intended to be open and flexible so that any part of this pipeline can be replaced with any other method with the same inputs and outputs in mind.

### 2.1 Anatomy generation module

The anatomy generation module creates an end-diastolic (ED) baseline label map. The myocardial labels are used as input of the cardiac motion module, as a shape to be deformed through time. The myocardial and surrounding tissue labels serve as input to the DENSE generation module, to generate the myocardium and background parts of the image. In this work, we used three types of anatomy generation methods, as outlined below, but it is intended to be fully flexible, to accommodate additional custom myocardial anatomies.

#### 2.1.1 XCAT phantom

The first option is to generate anatomies using the extended cardiac-torso (XCAT) tool [28], [29]. XCAT allows the generation of a whole-body phantom from a reference anatomy, which can be parametrized to generate variations in sex, heart shape, and cardiac cycle timings among other anatomical parameters. The spatial resolution, matrix size, field of view (FOV), and phantom rotation are also adjustable. While XCAT was originally designed for computed tomography simulations, it can also be used to generate a three-dimensional (3D) label map of tissues, or even 3D+time, as used in previous magnetic resonance imaging simulation studies [30], [25].

To generate short-axis labels, we considered that a baseline short-axis plane could be obtained with XCAT rotation parameters of 115^∘^, 35^∘^, and 240^∘^, respectively, around the x-, y-, and z-axes (these values are similar to the ones used in the MRXCAT study^2^ [30] and were empirically validated). The apex-base slice position can then be selected, where the range of potential slice positions is dependent on the pixel size. For instance, for a male base XCAT anatomy with 0.8 mm pixel size, the most apical segment would be around slice 1360, and the most basal segment around slice 1530, while for a base female anatomy, they would be, respectively, around slices 1260 and 1400. This module is particularly useful for creating variability in the apex-base direction in the baseline myocardial segmentation.

This module option is, with the current pipeline options, the only one that can generate background tissue anatomies, as can be seen in Fig. 1. However, as the analysis done in this study mainly focuses on strain results, the example datasets were generated using no background tissues to speed up the computation time.

#### 2.1.2 In-vivo DENSE image segmentation

A second option for the generation of ED anatomies is to use existing in-vivo DENSE images. A DENSE CMR dataset was acquired with informed patient consent and local ethical approval at the Royal Brompton Hospital in London from 2014 to 2021, as part of multiple research studies [31], [32], [33], [34]. Three hundred sixty short-axis acquisitions were acquired at 3T (MAGNETOM Skyra and MAGNETOM Vida, Siemens Healthineers AG, Forchheim, Germany) using a spiral cine DENSE research sequence [17], [40], [33]. This study cohort comprises both healthy individuals and patients with a range of cardiac conditions, including acute and chronic myocardial infarction, dilated cardiomyopathy (DCM), hypertrophic cardiomyopathy (HCM), myocarditis, recovered DCM, and sickle cell disease. We extracted manual ED myocardial segmentations from all short-axis segmentations. This creates a range of ED myocardial anatomies close to what we can expect in in-vivo studies, including anomalies such as those found in HCM and DCM patients.

#### 2.1.3 Annular computational phantom

Another option implemented for the anatomy generation module is the use of an annular phantom which can be used with computational models such as [35]. This phantom can be parameterized to generate annulus-shaped phantoms with user-selected endocardial diameter, epicardial diameter, and resolution.

This module creates more regular myocardial segmentations and can be useful to better understand the dynamics of the subsequent cardiac motion on the produced strain and DENSE images, without the variability due to irregularities in the anatomy.

### 2.2 Cardiac motion module

The cardiac motion generation module is used to generate myocardial deformations through time. These deformations have two purposes. On the one hand, they are used to generate 2D+time ground-truth pixel Green-Lagrangian strain maps, calculated using the means of isoparametric formulation with quadrilateral elements, as described in [36], [22]. On the other hand, Lagrangian displacements are then used as input for the DENSE simulation module to generate DENSE images. In the specific cardiac motion module implemented here, we use an ED myocardial mask which is deformed by the motion model.

This implementation of the cardiac motion module extends the work by Loecher et al. [27]. This method uses spatiotemporal second-order polynomial deformation fields applied to myocardial pixels in a contractile-like movement through time, assuring a broad range of locally smooth motion paths. We parametrized this method to randomly vary the radial dependency of the radial strain, from a constant radial strain in the transmural direction to an approximately linear dependency expected from incompressible computational models [35]. Additionally, an arbitrary rotation angle (2D twist) can be set to move the displacement vectors away from the center of the ventricle. Supplementary Fig. S1 shows that, in an incompressible annular computational phantom, the transmural variation of the radial strain is a second-order polynomial with the distance to epicardium, but is approximately linear over the thickness of the myocardium, with a higher strain at endocardium than epicardium. Strain map examples displaying different transmural radial strain patterns can be seen in Supplementary Fig. S2. Supplementary Fig. S3a shows the implemented distribution of the transmural gradient of the radial strain. The temporal dynamics followed a deformation through time with more motion at the beginning of the cardiac cycle than at the end, to reproduce what happens in-vivo (Supplementary Figure S3b). The simulated motion can be generated to only partially cover the cardiac cycle. Indeed, the DENSE sequence is prospectively triggered, so data around end-diastole might be missing in-vivo.

### 2.3 DENSE generation module

**Fig. 2:**
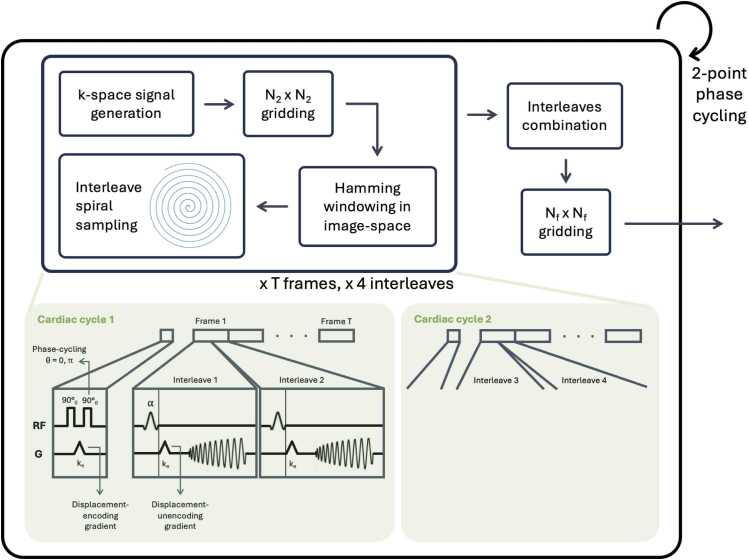
Illustration of the DENSE simulated acquisition. Synthetic displacements are used to generate k-space signal through time using a typical DENSE acquisition. The acquisition is repeated with a 2-point phase cycling and images are combined to produce 2D+time complex DENSE images. *DENSE* displacement encoding with stimulated echoes, *2D* two-dimensional, *RF* radiofrequency pulse

DENSE images were generated using a Bloch simulation. All the steps outlined below are summarized in Fig. 2.

The ED tissue labels obtained from the anatomy generation module in a grid of size (*N*_1_ × *N*_1_) were mapped to typical T1, T2, and M0 (equilibrium magnetization) values at 3T, for each pixel of the grid, considered as a spin isochromat. These values were estimated using normal values from previous 3T in-vivo CMR studies [37], [38] and empirically adjusted to match typical signal intensities found in in-vivo DENSE data [39]. The parameters corresponding to blood regions were manually adjusted to model dark blood, characterized by a rapid decay of the blood signal in the early cardiac phases due to the blood washout in DENSE. Using the pixel displacements generated from the motion module, these displacements were used in a simulation of a typical DENSE acquisition sequence to generate signal data at each temporal frame. The sequence consisted of a pair of 90-degree RF pulses with an encoding gradient of strength *k*_*e*_ = 0.1 cycles/mm between the pulses, played at the beginning of the cardiac cycle. The signal for imaging was generated with a variable flip angle RF pulse followed by an encoding gradient to match the first, as in in-vivo acquisition. The obtained transverse magnetization was then gridded onto an image of size (*N*_2_ × *N*_2_) using Fourier interpolation, with typically *N*_2_ < *N*_1_, which allows for the simulation of intravoxel dephasing. The obtained fully sampled Cartesian image was multiplied by a Hamming-shaped window in image space with a diameter at 0.5× of the maximum signal of 0.6× of the diameter of the image. This windowing step is used to approximate the in-plane excitation profile of the first two RF pulses (the 90-degree pulses) used in the typical DENSE protocol [40] which reduces the FOV contributing to the stimulated echo and avoids wrap from outside this region.

To simulate spiral sampling, we first generated DENSE images, fully sampled on a Cartesian matrix. Spiral k-space trajectories were then simulated using the sampling pattern typically used in-vivo (Fig. 2). Typical spiral DENSE sampling patterns use four interleaves acquired across two cardiac cycles, sampled in time as follows:

(1) $$\begin{array}{c} \forall l\in \left[1,2\right],\forall c\in \left[1,2\right],\forall i\in \left[0,T\right),\\ t_{lci}=t_{\mathrm{start}}+\epsilon_{c}+\frac{dt*\left(l-1\right)}{2}+dt*i, \end{array}$$

where *l* is the interleave index in each cardiac cycle, *t*_start_ is the time of the first imaging RF pulse (typically 15 ms); *ϵ*_*c*_ a random offset to simulate uncertainties in the detection of the R-wave of the electrocardiogram gating (set at *ϵ*_*c*_ ∈ [−5 ms, +5 ms], uniformly distributed), *c* being the cardiac cycle index; d*t* the time between each frame acquisition (typically 30 ms). This ensures that complementary interleaves are being sampled alternatively, for instance [15 ms, 45 ms, 75 ms] for frames 1, 2, 3 on interleave 1, and [30 ms, 60 ms, 90 ms] for frames 1, 2, 3 on interleave 2. The interleaves *S*(*t*) were then combined to form a fully sampled spiral pattern *S*_*i*_ so that:

(2) $$S_{i}=\underset{l\in \left[1,2\right],c\in \left[1,2\right]}{\sum }S\left(t_{lci}\right),$$

where each *S*_*i*_ corresponds to a frame with sampled time $t_{i}=t_{\mathrm{start}}+\frac{dt}{4}+dt*i$, typically [22.5 ms, 52.5 ms, 82.5 ms,…] in the ground-truth displacements. Frame interpolation can be optionally used, which is often used in-vivo to increase the reconstructed temporal resolution without increasing the acquisition time. In this case, additional frames $S\prime_{i}$ are generated to double the temporal resolution:

(3) $$S\prime_{i}=\sum_{c\in \left[1,2\right]}S\left(t_{\left\{l=1\right\}c\left(i+1\right)}\right)+S\left(t_{\left\{l=2\right\}ci}\right),$$

resulting in additional frames at typical times [37.5 ms, 67.5 ms, 97.5 ms,…].

Each sampled point of the interleave trajectories was also weighted using an inverse exponential decay over the course of the spiral readout to simulate T2* signal dephasing (using a T2* of 40 ms), and complex noise was added to the k-space to match a target myocardial signal-to-noise ratio (SNR). SNR in this study was calculated as the mean of the magnitude myocardial signal over the standard deviation of the magnitude signal in the same region.

Images were then generated using a non-uniform inverse fast Fourier transform, on a Cartesian matrix of size (*N*_*f*_ × *N*_*f*_), typically (90 × 90) or (128 × 128).

The pipeline is designed to support n-point phase cycling. The simulated cohort in this study was generated using 2-point phase cycling to match our current 2D in-vivo sequence [17]. The data from the two-phase cycles were combined into the final magnitude and phase images.

The different spatial encodings typically used in DENSE can be simulated by changing the encoding direction of the gradients. The final magnitude image is obtained by averaging the magnitude components of the x- and y-encoded acquisitions, and the final phase images are obtained by subtracting the x- and y-encoded phase from a 0-encoded phase image, as in typical in-vivo sequences [16].

### 2.4 Strain evaluation module

We computed the end-systolic (ES) Green-Lagrangian strain (circumferential and radial) using the open-source DENSEanalysis MATLAB tool (MathWorks, Inc Headquarters in 1 Apple Hill Drive, Natick, MA 01760, USA). A semi-automated version of DENSEanalysis as developed in [19] was used to process each case in under 30 s. DENSEanalysis implements spatial regularization of the extracted phase-image displacements to reduce the influence of the noise in the images on the calculated strain, using the MATLAB gridfit [41] function with a controlled level of smoothness. gridfit is used to estimate a Lagrangian displacement field from the Eulerian unwrapped displacement field at every frame, solving a minimization problem as:

(4) $${\mathrm{argmin}}_{L_{f}}\left(∥AL_{f}-E_{f}{∥}^{2}+k∥\nabla^{2}L_{f}{∥}^{2}\right),$$

where *A* is a sparse triangular interpolation matrix, *L*_*f*_ the Lagrangian displacement field, *E*_*f*_ the Eulerian displacement field, and the term in ∇^2^ is a Laplacian spatial smoothing term with a controlled level of smoothness k. A spatial regularization value of k = 0.9 or k = 0.8 is generally typical in DENSE studies [18], [33]. A polynomial model of order 10 for temporal fitting was used.

The DENSEanalysis strain and the ground-truth strain were compared using the average signed error (ASE) of the pixel strain at end-systole to estimate the biases introduced by regularization, as well as the standard deviation of the errors to estimate the capacity of the method to compensate for the image noise. Bland-Altman analyses were performed, comparing the analyzed global peak strain to the ground truth.

### 2.5 Virtual cohort generation

To evaluate the DENSE simulation pipeline, we generated a virtual cohort of 180 short-axis DENSE cines, using the above modules. One-third of cases were generated from XCAT ED hearts, one-third with in-vivo myocardial contours, and one-third with annular phantoms. We generated randomly positioned XCAT short-axis slices by tilting the short-axis plane by ±20^∘^, randomly rotating the image in-plane orientation, and randomly selecting the apex-base slice locations. Anatomies were generated at a resolution of 0.8 mm, for a matrix size of 240 × 240. The in-vivo myocardial contours were randomly selected from the patient/volunteer dataset of 360 cases and upsampled to 240 × 240. The upsampling operation is done by applying a Gaussian filter on the binary masks to smooth the edges, then spline interpolated to the target resolution and binarized using an intensity threshold of 0.5. An additional random dilation of up to six pixels was performed, given that manual myocardial segmentations on in-vivo DENSE images tend to generally be thinner than the underlying ground truth to avoid partial volume effects. The annular phantom cases were generated on a matrix of size 240 × 240, with an epicardial diameter ranging from 96 to 144 pixels, and an endocardial diameter ranging from 0.5× to 0.8× of the epicardial diameter.

The cardiac deformations and ground-truth strain maps were generated for R-R intervals ranging from 825 ms to 1200 ms, corresponding to 50–70 beats per minute. Frame times were sampled at t = 22.5 ms, 37.5 ms, 52.5 ms… when frame interpolation was turned on in the DENSE simulation pipeline, and t = 22.5 ms, 52.5 ms, 82.5ms… when frame interpolation was turned off, for 41–75 frames with frame interpolation, and 25–40 frames without frame interpolation. This range of reconstructed frames was empirically estimated from the in-vivo dataset characteristics. For each generated slice, a rotation was applied, randomly drawn from a uniform distribution [−0.5 rad, 0.5 rad] (peak-systolic rotation). The generated cardiac displacements were used in the DENSE simulation module, with *N*_1_ = 240, *N*_2_ = 60, and *N*_*f*_ ∈ {90, 128}, ensuring an underlying ×4 oversampling in the ground-truth displacement data compared to the generated DENSE phase displacements.

Each cine DENSE sequence was generated with noise to match a set target ES SNR, calculated over the magnitude signal of the myocardium. The ES magnitude SNR was either [3.9, 6.1] (low SNR, 60 cases, average SNR 5.2), [8.3, 11.5] (mid SNR, 60 cases, average SNR 10.1), or [13.9, 17.7] (high SNR, 60 cases, average SNR 15.3). This SNR range was empirically determined to match the in-vivo dataset statistics (in-vivo examples of DENSE images at different SNRs can be seen in Supplementary Fig. S4).

#### 2.5.1 Qualitative comparisons

We conducted a qualitative evaluation of the produced synthetic DENSE images. Synthetic images are compared to in-vivo data, and we displayed examples of synthetic images obtained by turning on/off various simulation features. The synthetic case examples are generated with a target myocardial SNR of 10, unless specified otherwise.

#### 2.5.2 Optimal DENSEanalysis spatial smoothing parameter estimation

We examined the influence of the spatial regularization parameter used in DENSEanalysis. The simulated DENSE cohort was used here to estimate an optimal spatial regularization on DENSEanalysis (as a smoothing value between 0 and 1) for different SNR levels.

## 3 Results

### 3.1 Qualitative comparison

**Fig. 3:**
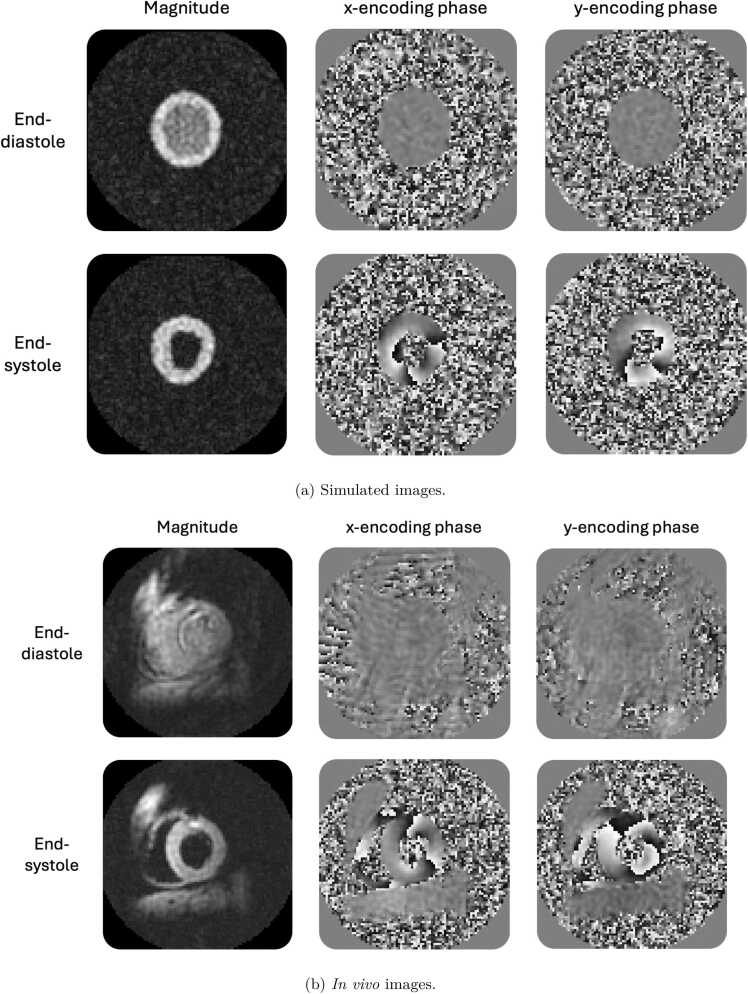
Visual examples of typical in vivo and simulation cases (magnitude and phase images). Similar phase wrapping patterns can be noticed, typical of DENSE displacement-encoding features. *DENSE* displacement encoding with stimulated echoes

Fig. 3 gives an overview of the simulated DENSE images, with an example of DENSE ED and ES magnitude and phase images from a synthetic case (Fig. 3a), in comparison with a typical in-vivo acquisition (Fig. 3b). The x- and y-encoding phases at end-systole display typical phase wrapping patterns, due to the intensity of the underlying myocardial displacements.

**Fig. 4:**
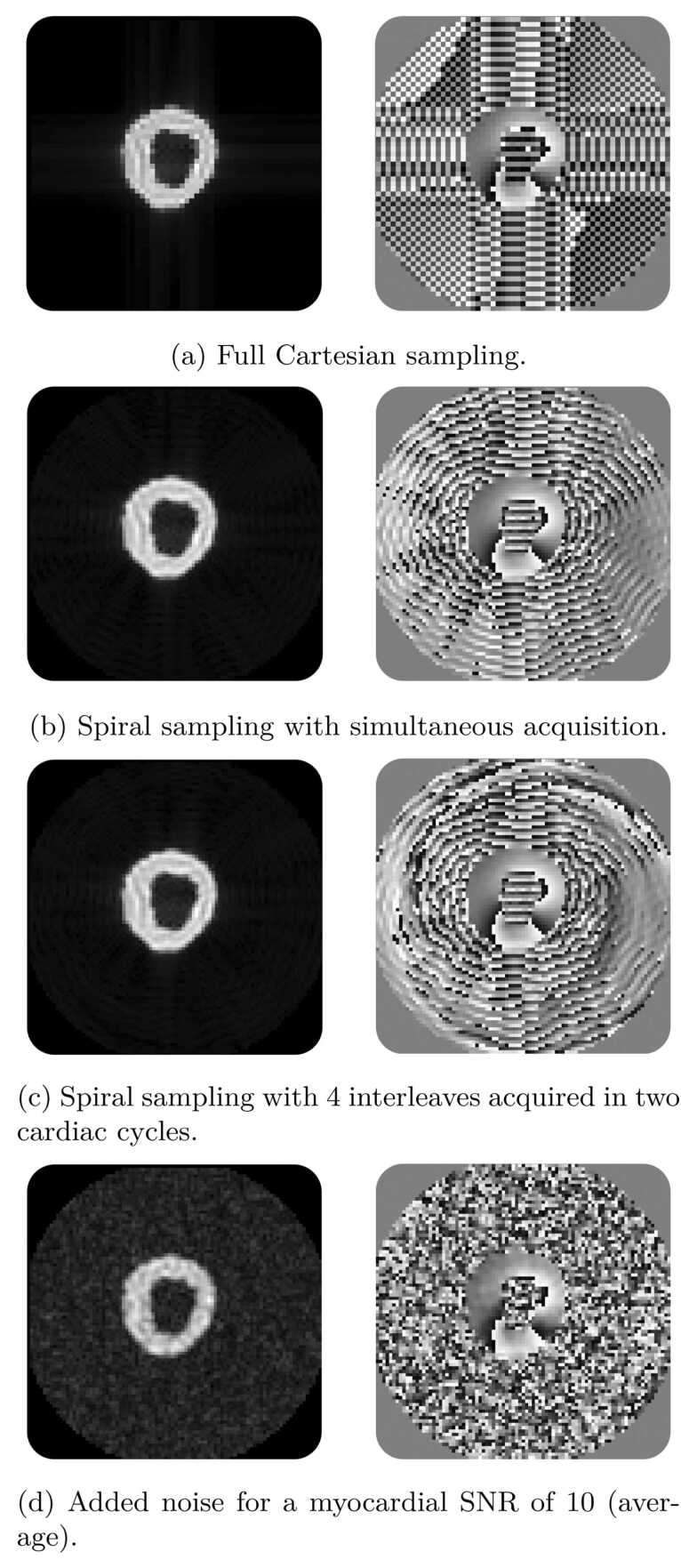
DENSE simulation examples of successive feature additions, from a fully Cartesian sampling without noise (a) to a spiral sampling (b) with 4 interleaves (c) and added noise for a target myocardial SNR of 10 (d). Left column: end-systolic magnitude image. Right column: end-systolic y-encoding phase image. Note: Small rounding differences between reference and encoded images can cause large phase changes in background regions with no signal. *DENSE* displacement encoding with stimulated echoes, *SNR* signal-to-noise ratio

In Fig. 4, we show the impact of the main features of the DENSE simulation module, for magnitude and phase images at end-systole. In Fig. 4a, a simulated DENSE reconstruction with a fully Cartesian sampled k-space without added noise; in Fig. 4b from the same synthetic case, reconstruction from spiral sampling with four interleaves, as if the spiral locations were all acquired simultaneously (limited artifacts) without noise; in Fig. 4c, reconstruction from spiral sampling with four interleaves, acquired in two cardiac cycles with sampling times similar to in-vivo acquisitions, without noise (displaying more artifacts); in Fig. 4d, with added noise at a typical SNR level (myocardial SNR of 10).

**Fig. 5:**
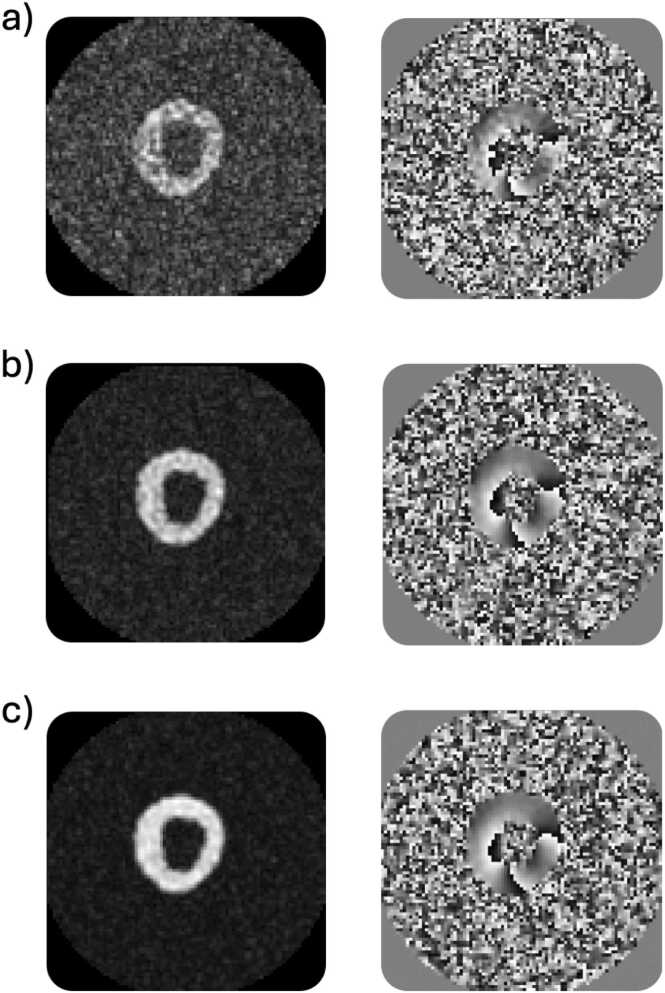
Visual impact of different SNR levels on the magnitude (left column) and x-encoding phase images (right column). SNR is calculated on the end-systole myocardial tissue signal. (a) low SNR of 5, (b) mid SNR of 10, (c) high SNR of 15. *SNR* signal-to-noise ratio

Fig. 5 displays the effect of noise in the k-space sampling, with three typical myocardial signal SNRs levels: low SNR, around 5, medium SNR, around 10, and high SNR, around 15.

**Fig. 6:**
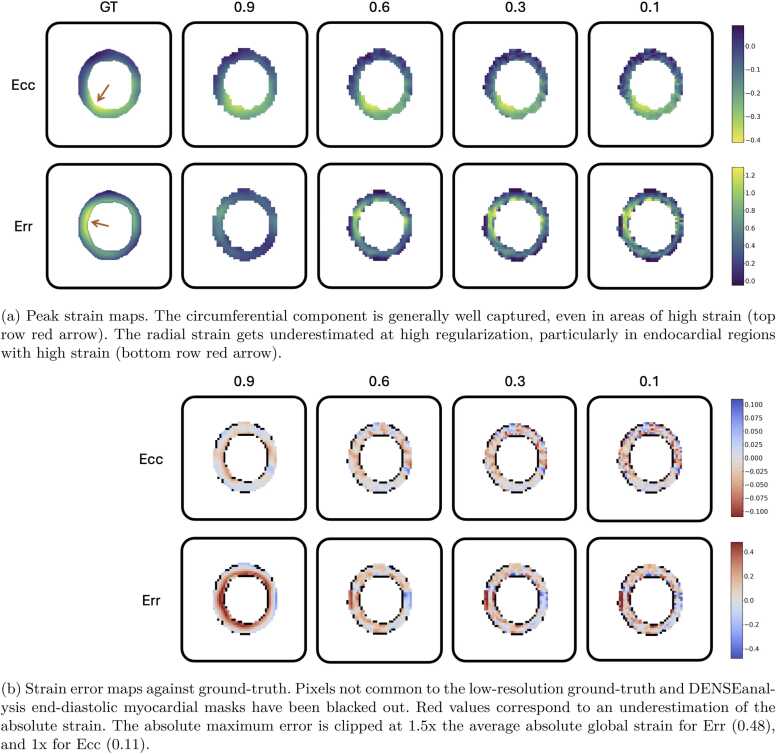
End-systolic strain maps of a typical simulated case. Left column: ground-truth strain. Subsequent columns: strain from DENSE images using DENSEanalysis with various spatial regularization parameters (0.9: high, typical / 0.1: negligible). *DENSE* displacement encoding with stimulated echoes, *Err* radial strain, *Ecc* circumferential strain

The same synthetic case was analyzed with DENSEanalysis, to extract the DENSE circumferential and radial strain components. The first two columns of Fig. 6 display the ES strain maps, for both the ground truth from the cardiac deformation module, and the strain obtained from the analysis of the DENSE images, using a typical spatial regularization setting in DENSEanalysis of 0.9.

**Fig. 7:**
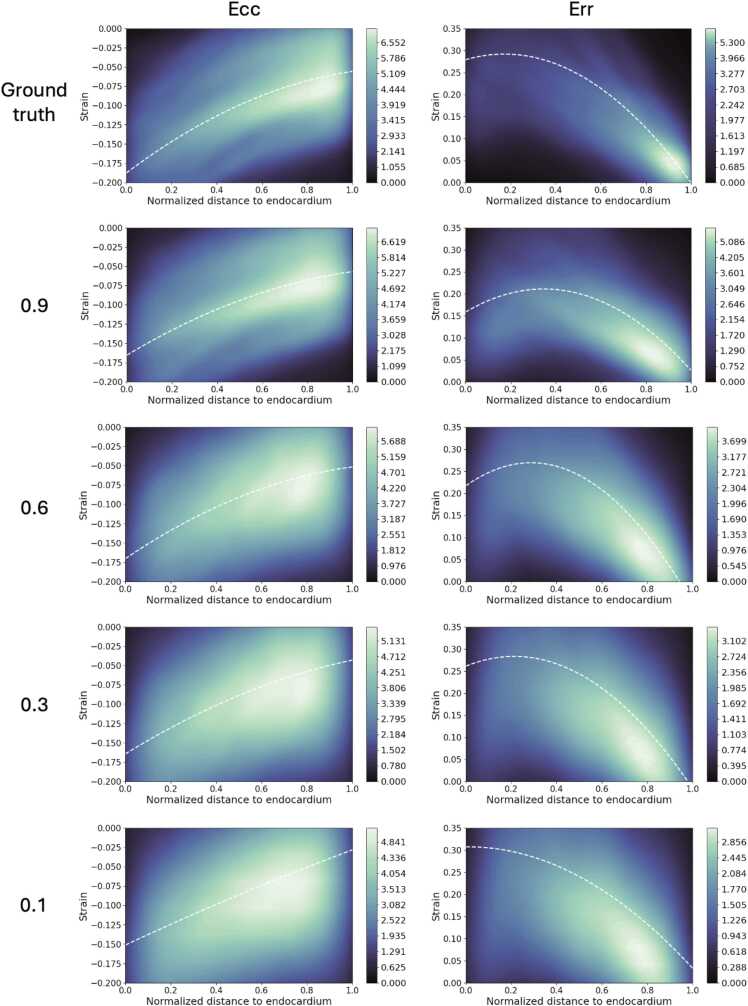
End-systolic transmural strain distribution over 180 simulated cases. Top row: ground-truth strain. Subsequent rows: strain from DENSE images using DENSEanalysis with various spatial regularization parameters (0.9: high, typical / 0.1: negligible). The white dashed line is the second-order polynomial regression model of the strain vs the distance to the endocardium. *DENSE* displacement encoding with stimulated echoes, *Err* radial strain, *Ecc* circumferential strain

DENSE strain data were also analyzed for the entire synthetic dataset of 180 cases at various SNR levels, with 60 being generated from XCAT contours, 60 from in-vivo ED segmentations, and 60 from annular phantom contours. The first two rows of Fig. 7 show the transmural distribution (from endocardium to epicardium) of the circumferential and radial strain components, for both the high-resolution ground truth and the DENSE strain obtained with DENSEanalysis using typical regularization (0.9). While the circumferential strain displays a similar transmural pattern to the ground truth, the radial strain displays a shifted transmural trend, particularly underestimated around the endocardium.

### 3.2 Qualitative analysis of DENSEanalysis performance

DENSEanalysis strain evaluation was performed under several spatial smoothing parameters, from 0.1 (i.e. low or negligible regularization) to 0.9 (high or typical regularization) with reference to the known ground-truth strain values. Fig. 6 shows the effect of the regularization on strain maps from an example synthetic case.

A more detailed analysis over the entire synthetic cohort can be found in Fig. 7. The effect of the regularization on the transmural variation of the radial strain is shown from endocardium to epicardium. The higher the regularization, the more the impact of the noise is reduced, but the strain is more underestimated, particularly around the endocardium.

### 3.3 Quantitative analysis of DENSEanalysis performance

We analyzed the pixel error of the ES radial strain. The ASE was 0.04 ± 0.09 for a typical regularization of 0.9, 0.02 ± 0.11 at 0.6, 0.00 ± 0.16 at 0.3, −0.01 ± 0.21 at 0.1. The ASE of the ES circumferential strain was −0.00 ± 0.04 for a typical regularization of 0.9, −0.01 ± 0.05 at 0.6, −0.01 ± 0.07 at 0.3, −0.01 ± 0.10 at 0.1. Table 1 summarizes these metrics and details the strain errors for different SNR levels.

**Table 1:** Strain pixel signed errors between the ground-truth and the DENSE strain at different regularization (k = 0.9 typical, 0.6, 0.3, 0.1 minimal) and SNR levels.

				Average signed errors (±std)	
	*k*	Low SNR	Mid SNR	High SNR	Global
Err	0.1	−0.03 ± 0.30	−0.00 ± 0.17	**0.01**± 0.14	−0.01 ± 0.21
	0.3	**0.00**± 0.20	**0.00**± 0.13	**0.01**± 0.13	**0.00**± 0.16
	0.6	0.04 ± 0.13	0.01 ± 0.10	**0.01**± 0.11	0.02 ± 0.11
	0.9	0.07 ±**0.11**	0.03 ±**0.08**	0.02 ±**0.09**	0.04 ±**0.09**
Ecc	0.1	−0.03 ± 0.14	−0.01 ± 0.06	−**0.00**± 0.05	−0.01 ± 0.10
	0.3	−0.01 ± 0.10	−0.01 ± 0.05	−**0.00**± 0.05	−0.01 ± 0.07
	0.6	−0.01 ± 0.06	−0.01 ± 0.04	−**0.00**± 0.05	−0.01 ± 0.05
	0.9	−**0.00**±**0.04**	−**0.00**±**0.02**	−**0.00**±**0.04**	−**0.00**±**0.04**

*DENSE* displacement encoding with stimulated echoes*, SNR* signal-to-noise ratio*, Err* radial strain*, Ecc* circumferential strain*, std* standard deviation

Bold values represent the optimal regularization level given average/std values per SNR split

**Fig. 8:**
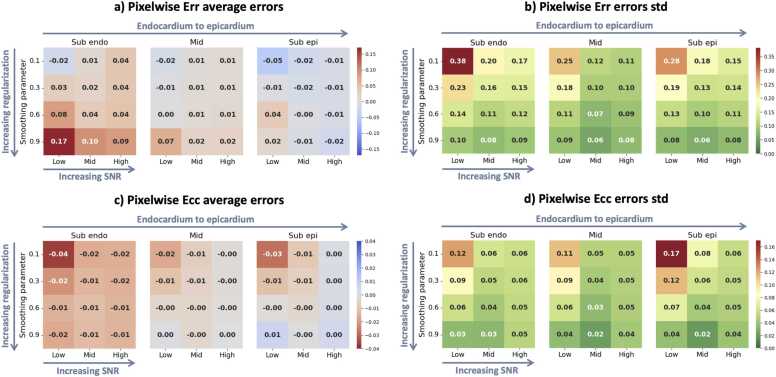
Strain pixel signed errors between the ground-truth and the DENSE strain at different regularization (0.9 typical, 0.6, 0.3, 0.1 minimal) and SNR levels, for 180 simulated cases. Top row: radial strain (Err). Bottom row: circumferential strain (Ecc). Left column: pixel average signed error. Right column: pixel signed error standard deviation (std). The values are aggregated over three transmural segments: subendocardial, mid-myocardial, and subepicardial. *DENSE* displacement encoding with stimulated echoes, *SNR* signal-to-noise ratio

A more detailed analysis can be found in Fig. 8, particularly showing the ASE for the subendocardial, mid-myocardium, and subepicardial transmural regions, at different regularization and myocardial SNR levels.

**Fig. 9:**
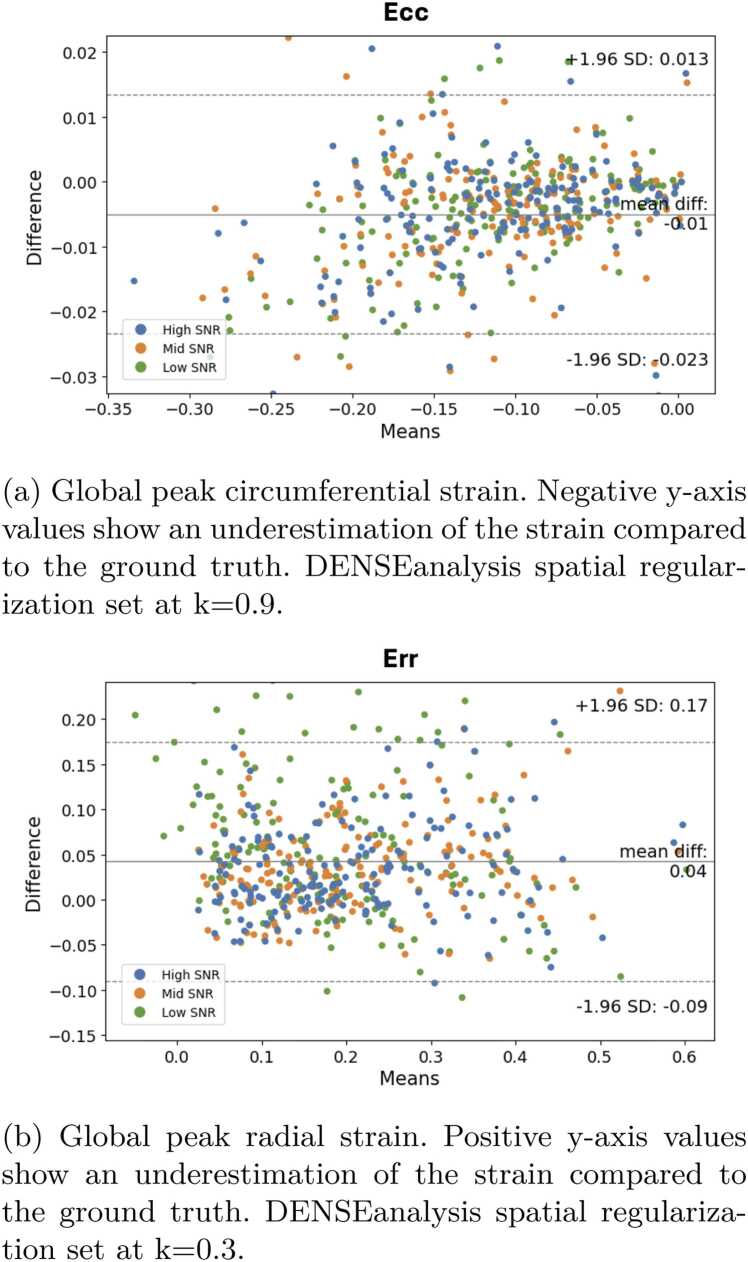
Bland-Altman plots of global peak strain, ground truth vs DENSEanalysis strain with ideal spatial regularization. *DENSE* displacement encoding with stimulated echoes, *Err* radial strain, *Ecc* circumferential strain

Fig. 9 displays Bland-Altman plots, analyzing the patterns of radial strain differences between the ground-truth strain and the strain obtained from DENSEanalysis (with a more optimal spatial regularization parameter of 0.3 for radial strain [Err] and 0.9 for circumferential strain [Ecc]). It shows more details about the distribution of the errors with respect to the underlying global peak strain. For Err (positive values represent an underestimation of the absolute strain), the bias was 0.04, with limits of agreement (±1.96 SD) of −0.09 to 0.17. For Ecc (negative values represent an underestimation of the absolute strain), the bias was −0.01, with limits of agreement (±1.96 SD) of −0.023 to 0.013.

## 4 Discussion

We developed a DENSE simulation pipeline, to generate realistic cine DENSE images with high-resolution ground-truth myocardial pixel strain. The DENSE simulation reproduces the majority of the key features of in-vivo cine DENSE acquisition, including phase cycling, variable flip angles, T2* decay during the readout, stimulated echo-reduced FOV imaging, interleaved spiral sampling, and variable SNR. Synthetic images show similar features to in-vivo cases, such as spiral artifacts and phase wrap. A synthetic dataset was generated using the developed pipeline and used to evaluate the strain accuracy of DENSEanalysis, one of the most widely used strain analysis tools for DENSE.

In our work, we noticed that current methods tend to underestimate strain values, particularly around the endocardial region. This is generally due to over-regularization, which is used to reduce the effects of the noise naturally present in DENSE images on the calculated strain values. This observation has been pointed out by previous studies [24], [39] and can be seen on the transmural strain plots in Fig. 7. Having an accurate ground-truth strain is key for quantifying the error in estimating pixel strain.

The study on DENSEanalysis shows that typical spatial regularization has a significant impact on the radial strain component in particular. As can be seen in Fig. 6, areas of high strain, indicated by the red arrows, are generally underestimated at high regularization, particularly for the radial strain component. As it is generally unfeasible to get ground-truth values for in-vivo strain to precisely quantify the regularization effect, creating a synthetic phantom with known ground truth is an ideal way to characterize strain errors. Most of the previous DENSE-based strain studies using DENSEanalysis have used a spatial regularization parameter of 0.8 or 0.9 [18], [24], [19], which, as we have seen in Fig. 8 and Table 1, is very likely to be sub-optimal for Err, even for noisy in-vivo data. A more optimal spatial regularization parameter can be determined by finding a trade-off between the global strain accuracy and the reduction of noise effects provided by regularization. Noise reduction and strain accuracy are maximized for Ecc using a regularization parameter of 0.9. The optimal parameter for Err varies between 0.6 and 0.3 for the tests used here, depending on the signal noise, with a variable transmural impact.

We note a slight increase in the systematic underestimation of the circumferential strain as the regularization decreases. While a decrease in systematic error with a stronger over-regularization may seem counter-intuitive, our analysis suggests that this originates from grid displacement fitting errors in the “gridfit” function used in the DENSEanalysis tool. To isolate the effect of DENSEanalysis, we analyzed the FIMH 2021 Kinematics Benchmark data with DENSEanalysis. The dataset consists of DENSE images from a cylindrical computational phantom, along with associated analytical ground-truth strain, as formulated in Supplementary 1. After running the analysis with various spatial regularization parameters, similar to the present study (k = 0.9, 0.6, 0.3, 0.1), we noted the same pattern in the impact of the regularization on the circumferential strain underestimation, indicating that it is not a feature of the simulation pipeline presented here but an effect of DENSEanalysis. This limitation motivates the use and development of different strain-fitting methods in future work, such as deep-learning modeling.

Additionally, gridding/resolution approximations can also impact the calculation of the strain from the displacement maps. As described in Section 2, strain is calculated using the means of isoparametric formulation with quadrilateral elements on the pixel displacements. In practice, this amounts to applying a central difference kernel on the immediately neighboring pixels of a target location. While this is used by DENSEanalysis, we also apply it to the high-resolution displacements ground truth to calculate the ground-truth strain in our simulation pipeline. As expected, the approximation errors from this method due to its discrete nature have a variable impact based on the resolution of the displacement maps. We validated that an ×4 resolution on the ground truth is optimal as a trade-off between limiting computational load and minimizing the discretization error on the computed strain. Details can be found in Supplementary 3.

The effect of regularization is likely to be influenced by various intrinsic features of the images, as we have seen here with the signal noise in the myocardium. Given known SNR levels, regularization, myocardial segments, and measured strain, we could attempt to correct the strain values based on the observed bias in a study like the present one. However, it seems more reasonable to consider moving away from deterministic regularization, to use data-driven regularization methods such as deep-learning methods to extract strain values directly from noisy images. The presented pipeline in this study will be used in future work to train such models in a supervised way, given the known ground-truth strain maps that can be generated alongside DENSE images, replacing the strain evaluation module with a deep-learning training process.

## 5 Limitations

Although the DENSE simulation module replicates most in-vivo acquisition features, some additional sources of artifacts are still yet to be implemented, such as B0 inhomogeneities and coil sensitivities, which can impact the signal distribution in the images. Moreover, the calculated in-vivo SNR used as a reference for the synthetic noise distribution is impacted by these factors, so that the match between in vivo and synthetic noise will be generally improved with these additional features. However, the current model probably uses higher noise in the synthetic images than necessary, as in-vivo images have stronger inhomogeneities in the signal distribution. We estimate the noise for our SNR calculations by using the standard deviation of the myocardial signal (due to in-vivo analysis limitations), but the impact of inhomogeneities in regional signal on the measured in-vivo SNR might be lower if alternative measures of SNR were used instead. However, the slight increase in noise in our simulations may be beneficial since deep-learning strain models using such synthetic images would develop stronger denoising capabilities. In future work, these additional features of the DENSE simulation module and their impact on the estimated strain will be explored.

While we engineered the cardiac motion module to have variable parameters for generating diverse deformation and strain distributions, we can see in Fig. 7 that more control and variability could still be added to the epicardial strain, given the high density of low radial strain in this region in the generated dataset. Similarly, the implemented deformations have a degree of variability with respect to the myocardial segments, as can be seen in the example of Fig. 6. The degree of variability is random in the current pipeline. It would be useful to make the change in strain between segments configurable, which would, for instance, enable the study of precise myocardial lesions on strain outputs. Since the proposed pipeline is modular, cardiac motion methods from other works [42], [25] can be added.

In this study, we analyzed the impact of regularization in DENSEanalysis, the most commonly used tool in clinical studies, over the estimated strain. This use case could be extended to methods that implemented other types of regularization, such as the recent work from Ghadimi et al. exploring combined spatiotemporal regularization [43].

We focused our analysis on ES strain results. A direct extension of this work would be to analyze the strain and strain rate accuracy through time, and in particular the impact of spatial and temporal regularization. To make this analysis relevant, it would be helpful to consider additional aspects in the temporal modeling of the cardiac motion simulation, including differences between systolic twisting and diastolic untwisting, rapid vs slow filling, etc. As can be seen in Supplementary Fig. S5, the temporal paths of the simulated displacements are ellipsoid. In reality, these paths are more irregular and differ between systole and diastole, as shown by Ghadimi et al. [43].

Future work should also extend the current pipeline to 2D long-axis and 3D displacements, taking through-slice displacements into account. 3D simulations would enable insights into 3D DENSE acquisitions [17] but require the extension of each module: 3D ED myocardial shapes would have to be created or acquired; the cardiac motion module would need to include apex-base motion and coherence of the in-plane displacements in the whole ventricle; the DENSE simulation module would need to support through-plane motion. Currently, we focused our development on generating and validating LV motion, and an extension to generate right-ventricular (RV) patterns would also be interesting [44]. The RV remains more challenging than left ventricular (LV) to simulate, given that its range of deformation is less well understood. The simulation of DENSE images including RV motion might be more challenging as well, given the thin nature of the compact RV and limited DENSE spatial resolution.

Additionally, the examination of DENSE strain analysis methods should investigate different regularization strategies than DENSEanalysis, for example using DENSE-SIM to train supervised deep-learning strain models with synthetic images.

## 6 Conclusion

In this work, we developed a simulation pipeline for the generation of realistic short-axis DENSE cine images, with ground-truth myocardial segmentation and strain. Such a pipeline enables accurate validation methods for strain analyses, whether to compare DENSE analysis methods, or understand the strain dynamics between different CMR modalities.

In particular, the pipeline can generate datasets for training deep-learning strain models in a truly supervised fashion. Further improvements in the accurate estimation of myocardial strain will enable clinical translation and improve outcomes for patients suffering with cardiovascular diseases.

## Funding

This work is funded by EPSRC Centre for Doctoral Training in Smart Medical Imaging (EP/S022104/1), by a Program Grant from the 10.13039/501100000274British Heart Foundation (BHF) (RG/19/1/34160), and 10.13039/501100011699Siemens Healthineers. The authors acknowledge financial support from the 10.13039/501100003921Department of Health (DoH) through the 10.13039/501100000272National Institute for Health Research (NIHR) comprehensive Biomedical Research Centre award to Guy’s & St Thomas’ NHS Foundation Trust in partnership with King’s College London and King’s College Hospital NHS Foundation Trust and by the NIHR MedTech Co-operative for Cardiovascular Disease at Guy’s and St Thomas’ NHS Foundation Trust. This work was supported by the 10.13039/100000002National Institutes of Health (NIH) [NHLBI R01 HL131823]. The views expressed are those of the authors and not necessarily those of the BHF, the NHS, the NIHR, the NIH, the DoH, or EPSRC.

## Author contributions

**Radhouene Neji:** Supervision. **Dudley J. Pennell:** Writing – review & editing, Project administration. **Yannick Brackenier:** Writing – review & editing, Methodology, Investigation. **Karl P. Kunze:** Writing – review & editing, Supervision. **Sonia Nielles-Vallespin:** Writing – review & editing, Supervision. **Andrew D. Scott:** Writing – review & editing, Supervision. **Daniel B. Ennis:** Writing – review & editing. **Hugo Barbaroux:** Writing – review & editing, Writing – original draft, Visualization, Validation, Methodology, Investigation, Formal analysis, Data curation, Conceptualization. **Michael Loecher:** Writing – review & editing, Methodology. **Alistair A. Young:** Writing – review & editing, Supervision.

## Ethics approval and consent

Data were acquired in accordance with protocols approved by all participating site’s institutional review boards for research involving human subjects, and all subjects provided informed consent.

## Consent for publication

No identifiable individual information was included in this study.

## Declaration of competing interests

The authors declare the following financial interests/personal relationships which may be considered as potential competing interests: Hugo Barbaroux reports financial support was provided by Siemens Healthcare Limited. Dudley J. Pennell reports financial support was provided by Siemens Healthcare Limited. Dudley J. Pennell reports financial support was provided by Cardiovascular Imaging Solutions. Karl P. Kunze reports a relationship with Siemens Healthcare Limited that includes employment. The other authors declare that they have no known competing financial interests or personal relationships that could have appeared to influence the work reported in this paper.

## Data Availability

The dataset used and analyzed during this study is available from the corresponding author upon reasonable request. The pipeline source code will be available at https://github.com/Hbarbaroux/DENSE_Sim/ after the publication of the present article.

## Footnotes

2 https://gitlab.ethz.ch/ibt-cmr-public/mrxcat-2.0.
