# Supplementary material for: DENSE-SIM: A modular pipeline for the evaluation of cine displacement encoding with stimulated echoes images with sub-voxel ground-truth strain
Source: J Cardiovasc Magn Reson. 2025 Feb 21;27(1):101866. doi: 10.1016/j.jocmr.2025.101866 (PMC12032873; doi:10.1016/j.jocmr.2025.101866)
Supplement: Supplementary file 1 — Supplementary material [file mmc1.pdf]

DENSE-SIM: A modular pipeline for the evaluation of cine DENSE images with sub-voxel ground-truth strain

Additional file 1

1 Transmural variations of strain on a cylinder

To understand the strain dynamics inside the myocardium, we can take a simple computational example. Strain components can be computed from the deformation gradient as follows:

$$E = \frac{1}{2}(F^T F - I), \tag{1}$$

where E is the strain matrix, and F is the deformation gradient.

The formulation also holds in cylindrical coordinates, where $\mathbf{E}_{rr}$, $\mathbf{E}_{cc}$ and $\mathbf{E}_{ll}$ are the diagonal elements of E when F is expressed in cylindrical coordinates F_{cyl}. Given a deformation field in cylindrical coordinates:

$$\begin{pmatrix} r \\ \theta \\ z \end{pmatrix} = f(R, \Theta, Z), \tag{2}$$

where R, Θ and Z are absolute coordinates in space, F_{cyl} can be expressed as [1]:

$$F_{cyl} = \begin{pmatrix} \dfrac{\partial r}{\partial R} & \dfrac{1}{R}\dfrac{\partial r}{\partial \Theta} & \dfrac{\partial r}{\partial Z} \\ r\dfrac{\partial \theta}{\partial R} & \dfrac{r}{R}\dfrac{\partial \theta}{\partial \Theta} & r\dfrac{\partial \theta}{\partial Z} \\ \dfrac{\partial z}{\partial R} & \dfrac{1}{R}\dfrac{\partial z}{\partial \Theta} & \dfrac{\partial z}{\partial Z} \end{pmatrix}. \tag{3}$$

As an example to derive the strain components with respect to their position (R, Θ, Z), let's consider a deformation field in cylindrical coordinates as defined by Perotti *et al.* in [2]:

$$\begin{aligned} r(R,\Theta,Z) &= a_1 + (1 + a_2)R + a_3R^2 \\ \theta(R,\Theta,Z) &= \Theta + a_4\frac{Z - Z_{\text{bot}}}{Z_{\text{top}} - Z_{\text{bot}}} \\ z(R,\Theta,Z) &= (1 + a_5)Z, \end{aligned} \tag{4}$$

where Z_{bot} and Z_{top} are respectively the bottom and top coordinates in mm of the cylinder, and a_i are computational deformation parameters defined as:

$$\begin{cases} a_1 = -21.301899 \\ a_2 = 0.875377 \\ a_3 = -0.009475 \\ a_4 = 0.079924 \\ a_5 = -0.141489 \end{cases} \tag{5}$$

Given the deformation field formulation, we can derive F_{cyl} as:

$$F_{cyl} = \begin{pmatrix} 1 + a_2 + 2a_3 R & 0 & 0 \\ 0 & \dfrac{a1}{R} + 1 + a_2 + a_3 R & \dfrac{a_4 \left[a_1 + (1 + a_2)R + a_3 R^2\right]}{Z_{\text{top}} - Z_{\text{bot}}} \\ 0 & 0 & 1 + a_5 \end{pmatrix}, \tag{6}$$

leading to a cylindrical expression of E as:

$$E = \begin{pmatrix} \mathbf{E}_{rr} & \mathbf{E}_{rc} & \mathbf{E}_{rl} \\ \mathbf{E}_{cr} & \mathbf{E}_{cc} & \mathbf{E}_{cl} \\ \mathbf{E}_{lr} & \mathbf{E}_{lc} & \mathbf{E}_{ll} \end{pmatrix}$$
$$= \frac{1}{2} \begin{pmatrix} (1 + a_2 + 2a_3 R)^2 - 1 & 0 & 0 \\ 0 & \left(\dfrac{a1}{R} + 1 + a_2 + a_3 R\right)^2 - 1 & 0 \\ 0 & \dfrac{a_4 \left[a_1 + (1 + a_2)R + a_3 R^2\right]^2}{R\left(Z_{\text{top}} - Z_{\text{bot}}\right)} & \left(\dfrac{a_4 \left[a_1 + (1 + a_2)R + a_3 R^2\right]}{Z_{\text{top}} - Z_{\text{bot}}}\right)^2 + (1 + a_5)^2 - 1 \end{pmatrix}. \tag{7}$$

Using the deformation parameters as defined in Eq 5, we can then plot the theoretical transmural variations of the circumferential and radial strain components, from endocardium ($R = 25$mm as in [2]) to epicardium ($R = 35$mm as in [2]), as shown in Figure S1.

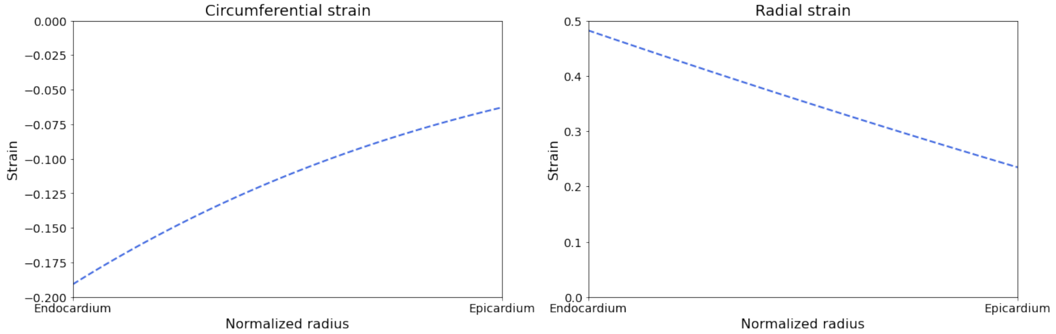

Figure S1: **Strain transmural variations.** Examples of circumferential (left) and radial (right) strain variations from endocardium to epicardium, from a cylindrical phantom.

2 Additional figures

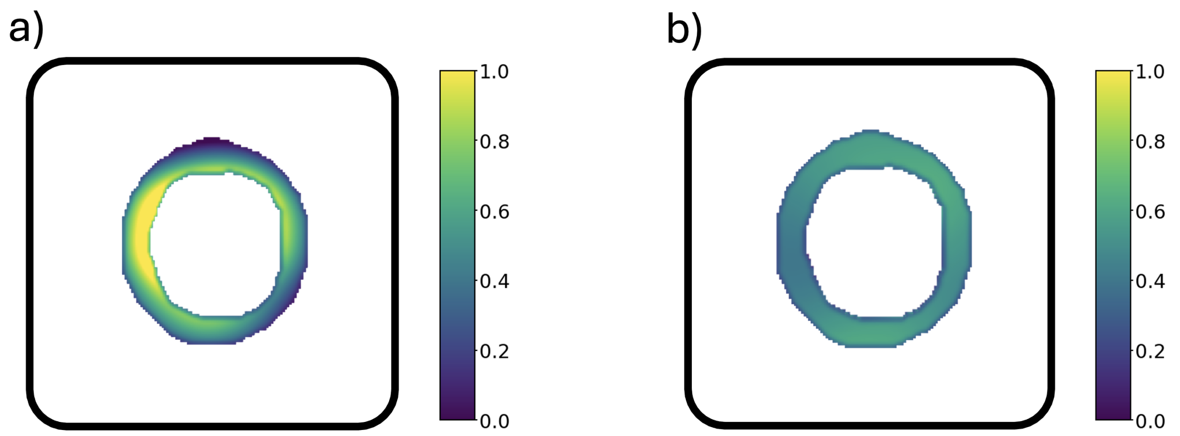

Figure S2: **Examples of high-resolution ground-truth radial strain at end-systole for simulated motion (x100 %).** a) Approximately linear strain transmural variation. b) No transmural strain variation.

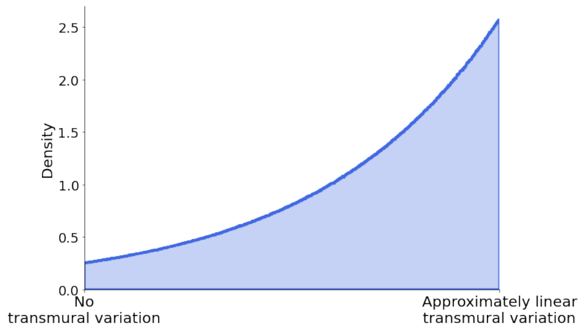

(a) Distribution of the random transmural variation of the radial strain, from no variation to approximately linear (higher strain at endocardium).

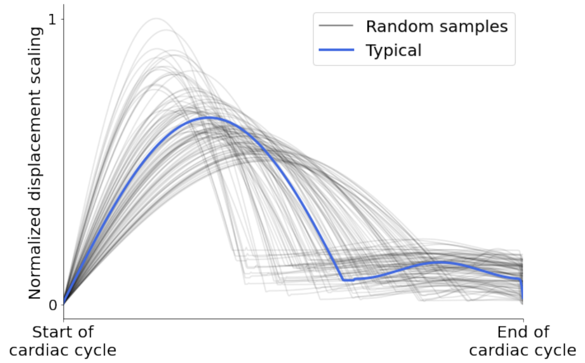

(b) Examples of deformation trajectories through time. 100 trajectories sampled (black), and median (blue).

Figure S3: Cardiac motion module parameter distributions.

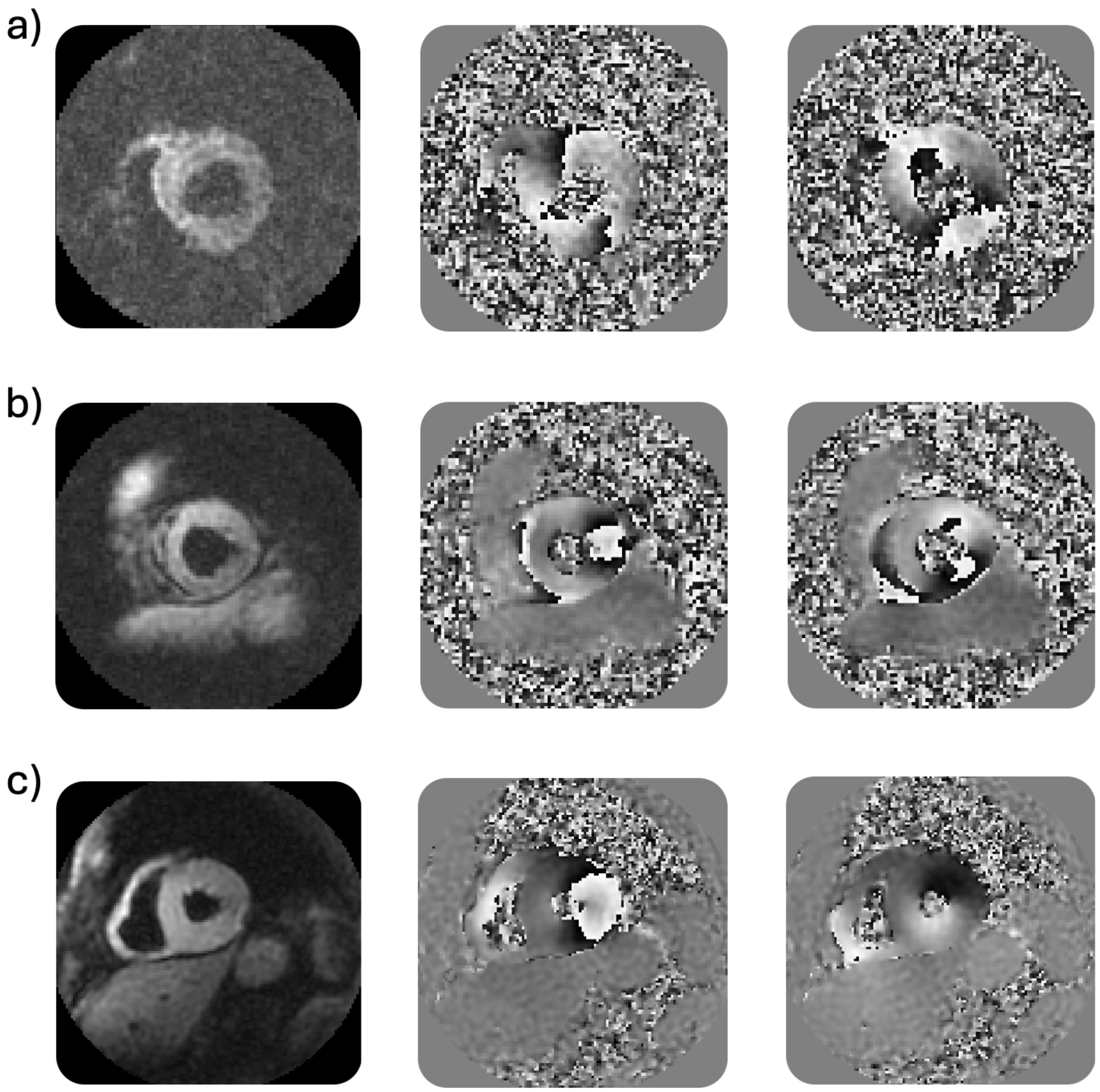

Figure S4: **Examples of *in vivo* DENSE images at different typical magnitude myocardial SNR levels.** a) Low SNR, about 5, b) Mid SNR, about 10. c) High SNR, about 15. Left column: magnitude, mid column: x-encoding phase, right column: y-encoding phase.

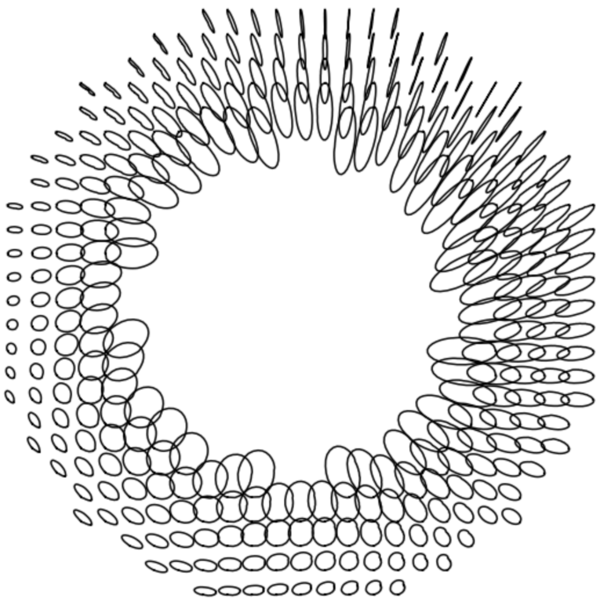

Figure S5: **Simulated displacements example.** The displacements follow ellipsoid paths in time, randomised through polynomial displacement fields and constrained to follow a contraction-like motion.

3 Impact of the resolution on strain calculations

DENSEanalysis [3, 4] computes strain from pixel displacements using the means of isoparametric formulation with quadrilateral elements. Concretely, the strain matrix E_i at every pixel i is calculated in Cartesian coordinates, and later converted to polar coordinates, where F_i is estimated from a least-square optimisation of the formulation $\partial X_i.F_i = \partial x_i$, with ∂X_i being a $(4, 2)$ matrix encoding the pixel coordinate differences between neighbouring pixels of i and i itself, and ∂x_i being a $(4, 2)$ matrix encoding the pixel displacement differences between neighbouring pixels of i and i itself. This can be seen as applying a central difference kernel covering direct neighbouring pixels on the coordinates and the displacements to calculate the elements $\frac{\partial x}{\partial X}$ for the deformation field F at every pixel.

It can then be induced that approximation errors might arise due to the discrete nature of the method. To understand its impact at different resolutions, we generated myocardial displacement maps on a high-resolution grid of size $(1920, 1920)$ from the DENSE-Sim cardiac motion module, corresponding to an x16 upsampling of a target resolution $(120, 120)$. Corresponding displacements were generated at x8, x4, x2, and x1 resolution, and the end-systolic strain was calculated with the method mentioned above. Figure S6 shows the relative global strain error as a percentage of the x16 resolution global strain. Displayed are the median relative errors and inter-quartile over 10 generated cases. Using these patterns, we considered that x4 oversampling was an appropriate ground-truth estimate for our study. Using the elbow method, such a target provides a reasonable compromise between reducing the impact of the approximation errors from the central difference kernel, and computation load. The median Ecc median error was 0.1% of the x16 global strain, and 1.2% for Err. Whether it is for calculating strain or later using the generated displacements for the DENSE simulation module of the pipeline, computations scale quadratically with the oversampling factor on average, given that the oversampling factor is applied in both directions of the image.

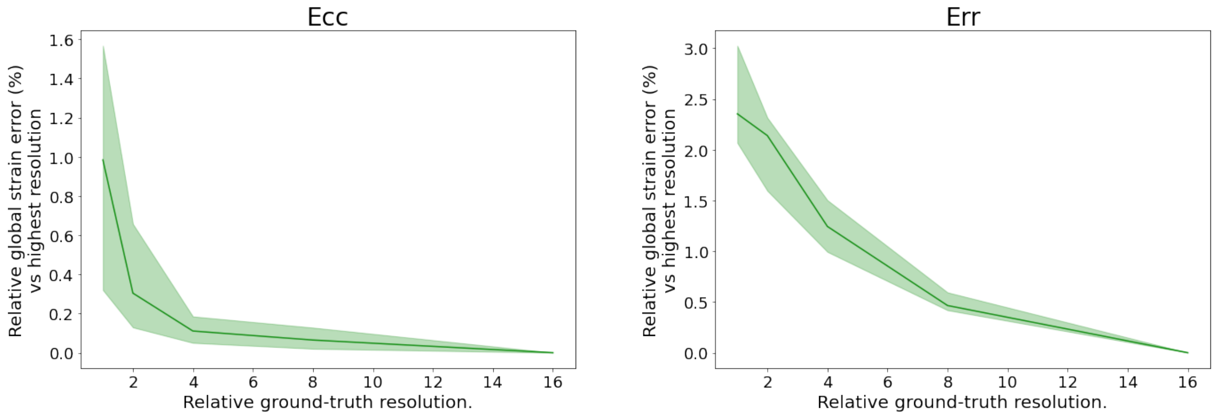

Figure S6: Relative errors (%) on the global end-systolic strain from displacement maps at different resolutions, where the highest resolution (x16) is considered the ground truth. Solid line: median over 10 cases. Shaded area: inter-quartile range. Increasing x-axis implies quadratically increasing computation time, while increasing y-axis implies increasing global strain error.

References

1. Andreas Klöckner. EN221 Summary;. Available from: `https://mathema.tician.de/dl/academic/notes/prelims/cmech.pdf`.
2. Luigi Perotti, Vicky Wang. FIMH 2021 - Cardiac Kinematics Benchmark; 2021. Available from: `https://fimh2021.github.io/assets/FIMH2021_Benchmark_v2.pdf`.
3. Spottiswoode BS, Zhong X, Hess AT, Kramer CM, Meintjes EM, Mayosi BM, et al. Tracking myocardial motion from cine DENSE images using spatiotemporal phase unwrapping and temporal fitting. IEEE Transactions on Medical Imaging. 2007;26(1):15–30.
4. Gilliam AD, Suever JD, and contributors. DENSEanalysis; 2021. Available from: `https://github.com/denseanalysis/denseanalysis`.
